# Commercial Motion Sensor Based Low-Cost and Convenient Interactive Treadmill †

**DOI:** 10.3390/s150923667

**Published:** 2015-09-17

**Authors:** Jonghyun Kim, Andrew Gravunder, Hyung-Soon Park

**Affiliations:** 1 DGIST, Department of Robotics Engineering, 333 Techno Jungang-daero, Hyeonpung-Myeon, Dalseong-gun, Daegu 42988, South Korea; E-Mail: jhkim@dgist.ac.kr; 2 National Institutes of Health, Rehabilitation Medicine Department, 10 Center Drive, MSC-1604, Bethesda, MD 20892, USA; E-Mail: andrew.gravunder@nih.gov; 3 Korea Advanced Institute of Science and Technology, Department of Mechanical Engineering, 291 Daehakro, Yuseong-gu, Daejeon 34141, South Korea

**Keywords:** motion sensor, interactive treadmill, low-cost, convenience, self-paced treadmill, treadmill-on-demand, walking, locomotion

## Abstract

Interactive treadmills were developed to improve the simulation of overground walking when compared to conventional treadmills. However, currently available interactive treadmills are expensive and inconvenient, which limits their use. We propose a low-cost and convenient version of the interactive treadmill that does not require expensive equipment and a complicated setup. As a substitute for high-cost sensors, such as motion capture systems, a low-cost motion sensor was used to recognize the subject’s intention for speed changing. Moreover, the sensor enables the subject to make a convenient and safe stop using gesture recognition. For further cost reduction, the novel interactive treadmill was based on an inexpensive treadmill platform and a novel high-level speed control scheme was applied to maximize performance for simulating overground walking. Pilot tests with ten healthy subjects were conducted and results demonstrated that the proposed treadmill achieves similar performance to a typical, costly, interactive treadmill that contains a motion capture system and an instrumented treadmill, while providing a convenient and safe method for stopping.

## 1. Introduction

Treadmills provide safe, reliable, and steady-state linear paths for fixed-speed walking, and, therefore, have been widely used in biomechanical, physiological, and clinical research to investigate walking in humans. However, several studies have reported that treadmill walking (TW) alters walking performance when compared to walking in daily life, usually referred to as overground walking (OW) [1,2,3,4]. The mostly commonly cited reasons for these differences include limited length of the treadmill belt, fixed belt speed, and the absence of visual flow [5]. Of those, the first two reasons compel fixed-speed walking on the treadmill.

In order to make TW more similar to OW, a novel, interactive treadmill [6] (ITM, also called treadmill-on-demand [7] or self-paced treadmill [5,8]) was proposed. The ITM consists of sensors for measuring the subject’s position, a controllable treadmill including a low-level belt-speed controller, and a high-level control scheme for keeping the subject within the desired area of the treadmill belt while allowing the subject to freely accelerate or decelerate at will. Since the ITM allows the subject to walk a long distance while naturally varying their walking speed, it effectively addresses the limited length of the treadmill belt and fixed belt speed limitations that result in differences between TW and OW.

**Figure 1:**
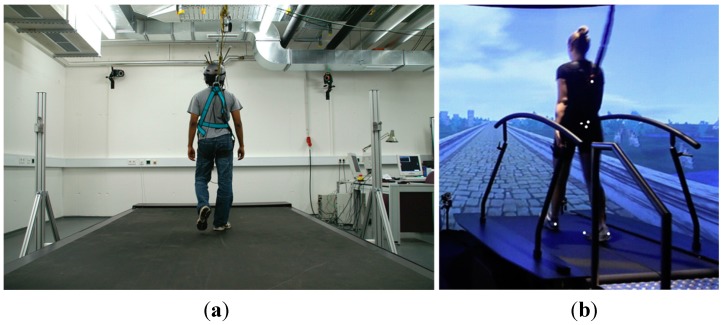
Specialized high-cost treadmills have been used in previous interactive treadmills (ITMs). (**a**) Large custom-made treadmill; (**b**) Instrumented treadmill.

There have been several reported attempts at implementing the ITM. However, to our knowledge, all existing attempts have required expensive equipment (*i.e.*, expensive sensor system and/or expensive treadmill) and sophisticated setup procedures prior to treadmill walking. For example, a commercial motion capture system (Mocap) is most commonly used to continuously monitor the subject’s body position, but it is expensive [5,6,9,10,11,12,13]. Moreover, Mocap requires additional setup procedures, such as attaching either passive or active markers to the proper locations on the subject’s body. One study eliminated the setup procedures by using force plates in an instrumented treadmill to measure the subject’s position [14], but the high cost of the treadmill is prohibitive. In other cases, less expensive sensors, such as an ultrasonic range finder [7], potentiometer [8], and force sensors [15,16] were used, but these require the attachment of a rigid harness to the subject, causing both inconvenience and contributing to an unnatural feeling while walking. Regarding the treadmill itself, most previously reported attempts have used specialized high-cost treadmills (Figure 1), such as custom-designed [8,12,15] or instrumented treadmills [5,6,9,10,11,14], for high-control performance. The ITMs based on these high-cost treadmills (>100 kUSD) are effective, but the cost prevents them from being used widely and in most clinical environments.

As mentioned above, the major limitations (cost and sophisticated setup requirements) associated with previously developed ITMs were related to the sensor component used to measure the subject’s position on the treadmill. Recently, novel motion sensors, such as Microsoft Kinect (Microsoft Corporation, Redmond, WA, USA), originally developed for gaming applications, have been applied to both biomechanical [17,18] and clinical studies [19,20]. These infrared-type motion sensors are a promising way to overcome ITM limitations because they are cost-effective, non-contact sensors that can measure the three-dimensional position of anatomical landmarks without lengthy setup [17,18,19,20]. Moreover, gesture recognition by the sensors can also be used as a non-contact interface with which a subject may conveniently operate an ITM.

This paper proposes and evaluates a low-cost and convenient sensor system for ITM which ultimately aims at broader research and clinical use. For this purpose, a commercial infrared-type motion sensor with corresponding control system was proposed and its performance was evaluated in comparison with the ITM using the expensive Mocap system. The sensor, which is very similar to the Microsoft Kinect, is inexpensive, and has negligible setup time because it does not require the placement of active/passive markers on anatomical landmarks. Moreover, it contains a gesture-recognition function that enables the subject to safely and conveniently stop the ITM. In addition, a high-level belt speed control scheme was implemented with a commercial-grade treadmill to simulate OW in a manner far less expensive than custom/instrumented treadmills. A pilot experiment with ten healthy subjects was conducted to qualitatively and quantitatively evaluate the performance of the proposed ITM. In this paper, we hypothesized that (1) the performance of the proposed ITM would be comparable to that of a typical ITM, which uses Mocap and an instrumented treadmill and (2) the proposed ITM would achieve acceptable level of safety due to the gesture-based stopping method developed.

## 2. Proposed Interactive Treadmill

**Figure 2:**
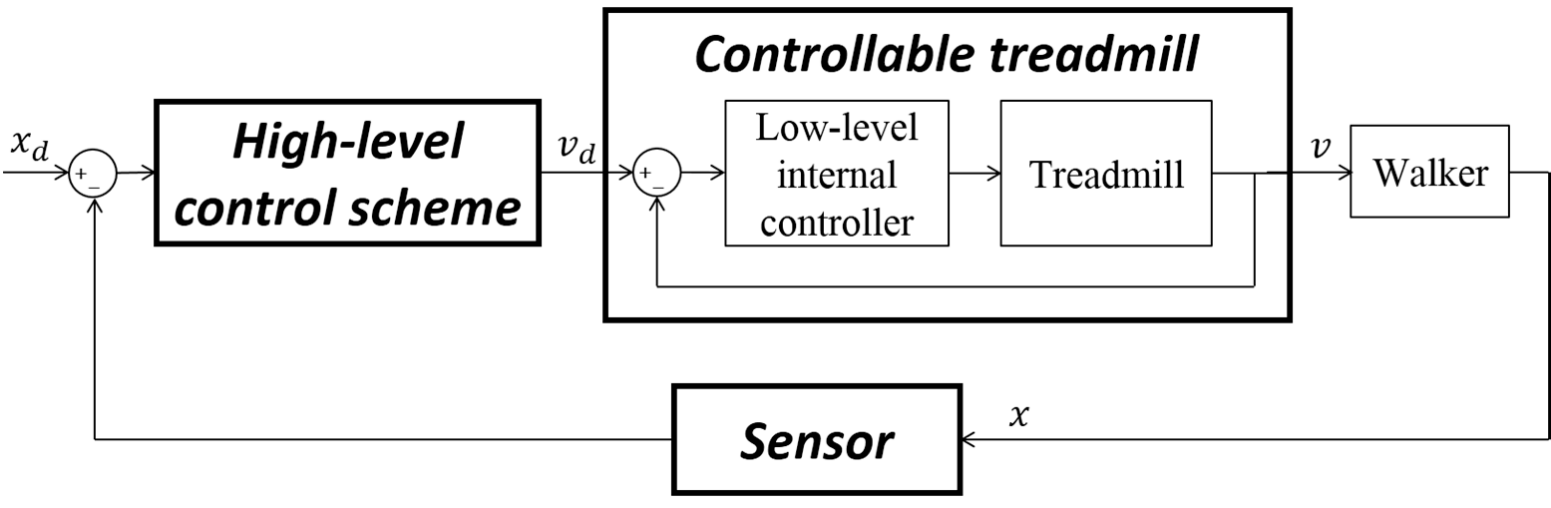
Description of the interactive treadmill.

To achieve the function of an ITM, the speed of the treadmill belt should automatically and dynamically adapt to keep the subject within the bounds of the treadmill. For instance, if the subject is located at the front of treadmill, the speed of the belt should increase to adjust the subject’s location backward. Thus, an ITM is a feedback-controlled system that operates via a sensor and high-level control scheme, as illustrated in Figure 2. Based on the subject’s sensed position (x), the control scheme generates a desired belt speed ($v_{d}$) that becomes the input for the low-level internal controller of a controllable treadmill, and based on that input the internal controller adjusts the belt speed (v) to the desired level. Here, the desired position ($x_{d})$ needs to be determined within the treadmill.

### 2.1. Sensor Component: Motion Sensor

**Figure 3:**
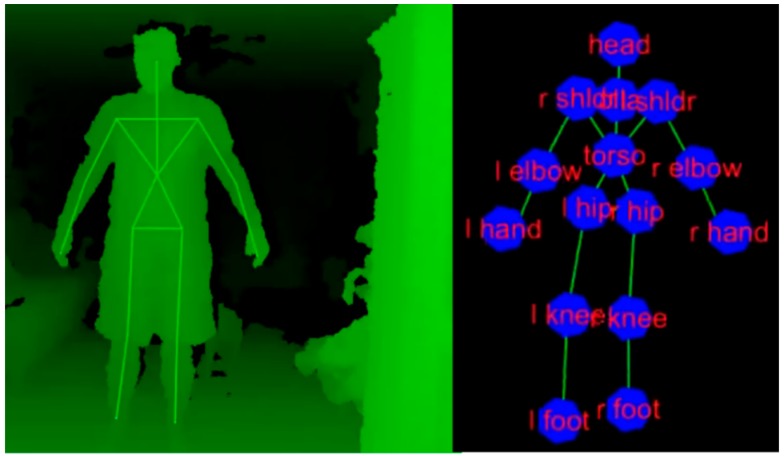
Skeleton model and landmarks of Xtion motion sensor [22].

A core idea of the proposed ITM is to apply a low-cost and non-contact motion sensor, the ASUS Xtion (ASUSTeK Computer Inc., Taipei, Taiwan), as the sensor component of the ITM. Similar to the Microsoft Kinect, Xtion has infrared units that generate a three-dimensional (3D) depth map of its surroundings and contains an adaptive algorithm in order to automatically acquire typical human anatomical landmarks (fifteen landmarks) on the body [21]. A previous study has demonstrated that the accuracies of the Xtion sensor and the Kinect sensor are comparable [21]. The Xtion has two additional advantages: adjustable speed and support of open-source drivers. The Xtion provides two modes with different sampling frequencies: a VGA (640 × 480) normal mode with a 30 Hz sampling frequency and a QVGA (320x240) fast mode with a 60 Hz sampling frequency, allowing the user to choose the appropriate mode for their specific application. The customizable open-source drivers, OpenNI and NITE, easily allow Xtion to measure the 3D positions of landmarks based on a model human skeleton, as illustrated in Figure 3.

The ITM sensor should be able to obtain the subject’s anterior/posterior position on the treadmill belt, along the walking direction. Since the torso is one of the most stable anatomical parts (e.g., the torso does not fluctuate much during walking) and the “torso” landmark is the most accurate and reliable landmark available with the Xtion sensor, we opted to track the “torso” landmark (Figure 3) and used its one-dimensional (1D) position data in the anteroposterior axis to obtain the subject’s position on the treadmill. The “torso” for the Xtion is defined as the intersection of two lines: a line from the right shoulder landmark to the left hip landmark and a line from the left shoulder to the right hip (Figure 3). In addition, the Xtion can acquire the “torso” landmark even if many of the distal landmarks (e.g., hand, elbow, foot, and knee) are lost. It is noteworthy that those distal landmarks are less accurate due to the limitations of the adaptive algorithm of the sensor [23].

Compared with previous expensive and/or inconvenient sensors used for an ITM, the retail price of the Xtion sensor is less than 200 USD and the system enables measurement of landmark positions without additional procedures, such as placing markers or donning a rigid harness. Hence, the Xtion is an appropriate motion sensor for the proposed ITM to reduce cost and improve convenience.

### 2.2. Treadmill and Control Components

One aim of this paper is the low-cost implementation of an ITM for widespread use. Therefore, the proposed ITM needs to consider the use of an inexpensive treadmill. However, to our knowledge, most previously reported ITMs have used specialized high-cost treadmills, such as an instrumented treadmill that contains force plates (Figure 1b) or a large custom-made treadmill (Figure 1a). These treadmills add the following benefits:

(1) The force plate of the instrumented treadmill can be used to estimate the subject’s position without an additional sensor that would require an inconvenient setup [14].
(2) The long belt length of the custom-made treadmill makes implementing an ITM easier by reducing the difficulty of keeping the user within the bounds of the treadmill belt [12].
(3) In general, a high-cost treadmill contains a higher-quality low-level internal controller (large maximum acceleration rate, fast sampling rate, and high-speed resolution) than an inexpensive treadmill. Note that the internal controller in a controllable treadmill is different from the high-level control scheme for implementing an ITM (Figure 1). Table 1 represents a typical comparison of the internal controllers of each type of treadmill.

**Table 1:** Different characteristics of internal controllers in treadmills [13].

	Inexpensive Treadmill	Specialized High-Cost Treadmill (Instrumented Treadmill)
Maximum acceleration rate (m/s^2^)	2	>20
Sampling rate (Hz)	8	>120
Speed resolution (m/s)	0.045	<0.001

The choice of the Xtion motion sensor eliminates the first benefit of an instrumented treadmill. However, the other benefits of a high-cost treadmill remain valid and there is a potential for degraded performance of the proposed ITM with an inexpensive treadmill.

To achieve better performance with an inexpensive treadmill, the high-level control scheme needs to be chosen carefully. The most popular choice is a proportional-integral-derivative control (PID) [7,8,13]. However, an ITM based on PID and an inexpensive treadmill could cause subject instability due to a low maximum acceleration rate, short belt length, and a slow sampling rate. The use of PID requires a fast belt acceleration rate, especially with a slow sampling rate [6,7,13]. Thus, it can cause a windup phenomenon [24] with the low-level internal controller of an inexpensive treadmill. Moreover, a shorter belt length requires a faster acceleration rate because only a short walkway distance is available to adjust belt speed [12]. Recently, a novel control scheme was proposed for an ITM by using a Mocap that has sub-millimeter accuracy and its performance in simulating OW has been verified through comparison with several existing control schemes, including the PID [6,12]. In the novel control scheme, a pelvic position controller with a dynamic observer does not contain any integral action and, therefore, it only requires a slow belt acceleration rate. In addition, its variable reference algorithm effectively increases belt length. Therefore, for the ITM proposed here, this novel control scheme was chosen as it can be used with an inexpensive treadmill. The details of the control scheme have been well documented [6,12].

### 2.3. Convenient and Safe Stop Based on a Non-Contact Interface

In practice, the ability of a subject to safely stop the treadmill of an ITM is an essential function. Previous ITMs have not thoroughly addressed this function, because the high-level control schemes used in previous models theoretically would be able to position the subject on a pre-defined treadmill location within a short time duration—thus executing a quick and safe stop. However, this is only feasible with an expensive setup (*i.e.*, a long belt length and/or large belt acceleration are required to safely regulate the subject’s position during a stop because stopping represents a sudden change in speed to zero). Since the proposed ITM cannot make use of the same features used with expensive equipment, an alternative solution to the problem of stopping was developed.

One existing solution required the subject to hold a switch to send his/her intention to stop to the ITM. This solution, however, is unnatural and inconvenient, especially for an ITM because TW on an ITM can be much more dynamic than conventional TW. Thus, for convenient and safe stopping, we developed a non-contact interface between the subject and the treadmill using the Xtion sensor. Since the Xtion can recognize gestures by tracking anatomical landmarks, it can also be used as a non-contact interface. In this paper, we chose a “hand raise” gesture to indicate a stop. The following method was developed by trial and error method, and the treadmill detects the subject’s intention to stop when the following conditions are met for a 1-s interval, the ITM will detect the subject’s intention to stop.

(1) On the left or right side, the location of the hand landmark was superior to the elbow landmark and the absolute velocity of the hand landmark was smaller than 0.01 m/s.
(2) The hand of the first condition is not located within 0.2 m of the head landmark.

Note that the second condition distinguishes a common gesture, such as head touching or scratching, from the defined “hand raise.” After detecting the “hand raise” gesture, the proposed ITM automatically quits the high-level control scheme, and goes into “cool-down” mode, which monotonically decreases belt speed. Thanks to the non-contact interface, conveniently activated by the subject’s gesture, the proposed ITM is able to achieve a safe stop without expensive setup requirements.

### 2.4. Implementation of Proposed ITM

**Figure 4:**
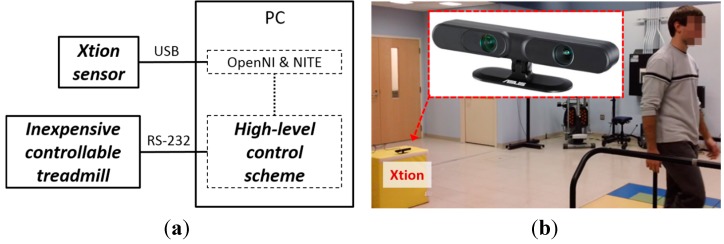
Implementation of proposed ITM. (**a**) Component combination (**b**) Location of Xtion motion sensor [22].

The ideas above show that a low-cost and convenient ITM can be developed by applying an Xtion sensor and the high-level control scheme to an inexpensive, controllable treadmill. Thus, we implemented the ITM, as illustrated in Figure 4a. The Xtion sensor was connected to a PC via USB and OpenNI and NITE software drivers were used to capture the subject’s position. The high-level control scheme used to calculate desired belt speed was implemented using a C++ custom program and the desired speed was sent to the low-level internal controller of the controllable treadmill via RS-232 communication. It should be noted that the inexpensive treadmill for the proposed ITM was simulated on an instrumented treadmill (Bertec Corporation, Columbus, OH, USA) by artificially reducing its performance (Table 1) through a custom C++ program: the belt acceleration speed was limited to 2 m/s^2^; the desired belt speed command was adjusted to 0.045 m/s resolution, and the sampling rate of the treadmill was reduced to 8 Hz.

The Xtion was operated in normal mode with a 30 Hz sampling frequency, because the sampling rate in the proposed ITM is limited by the simulated internal controller of the treadmill (8 Hz). It is worth mentioning that the fast mode (60 Hz) performs better with high-cost treadmills that have low latencies. The Xtion sensor was located at 2 m behind of the end of treadmill belt and at a 0.8 m height above the ground in order to allow visualization of the subject’s entire body (Figure 4b).

## 3. Methods

### 3.1. Experimental Design

Though the main contribution of the proposed ITM is to reduce cost and to be convenient, it should also have acceptable performance characteristics for an ITM (*i.e.*, it should make TW similar to OW). In order to investigate the possible performance degradation caused by the low-cost motion sensor and inexpensive treadmill, a comparison study was conducted between the proposed ITM and a typical ITM.

The typical ITM, which is similar to the system previously reported [5,6,9,10,11], incorporated a commercial Mocap (Vicon Motion Systems Ltd., Oxford, UK) and an instrumented treadmill (Bertec Co., Columbus, OH, USA). Note that the proposed ITM also used this instrumented treadmill in order to simulate an inexpensive commercial treadmill by artificially limiting its performance. Using the Mocap, the typical ITM can acquire the subject’s position at 120 Hz (four times faster than the proposed ITM). Moreover, the low-level internal controller of the instrumented treadmill can accept the speed command of the high-level control scheme at 120 Hz (fifteen times faster than the proposed ITM). Attaching passive markers is essential for implementing the typical ITM while the proposed ITM did not require any markers. In the proposed ITM, the torso landmark of Xtion is the best option for measurement of the subject’s position, as mentioned in Section 2.1. However, the raw data of the landmark still contains greater noise than the Mocap, due to random errors in detecting the landmark. According to the noise magnitude of the Xtion sensor in previous report [21,25], the maximum speed error caused by the sensor noise is 26 mm/s which is negligible compared to natural fluctuation of walking speed in human walking [11]. In order to attenuate the noise, the filtered raw data was used for calculating desired belt speed after filtering with a second-order Butterworth low-pass filter with a 10 Hz cutoff frequency.

**Figure 5:**
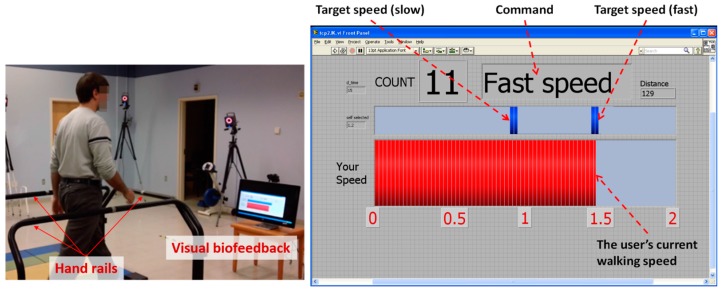
Experimental setup: handrails and visual biofeedback [22].

For fair comparison, both ITMs use the same high-level control scheme with identical control parameters, as summarized in Table 2. Note that the detailed description of the control parameters has been well documented [6,12]. Moreover, a visual biofeedback for walking speed that displayed the subject’s walking and target speeds on a PC monitor in real-time was developed using a custom-built program (LabVIEW, National Instruments Corp., Austin, TX, USA) (Figure 5). Since the two ITMs being compared used the same treadmill, subjects could be blinded to the identity of the ITMs throughout the experiment.

In addition, we investigated the stop function of the proposed ITM using the Xtion sensor. Handrails were installed on the front and two sides of the treadmill as a safety measure for the subject, especially for the stop test (Figure 5).

**Table 2:** Control parameters of high-level control scheme [22].

$k_{pos}$	$k_{a}$	$k_{obv}$	**** $k_{ref}$
2.0	2.0	3.0	0.4

### 3.2. Protocols

Ten healthy subjects (five males) with a height range of 153–182 cm (168.4 ± 10.2 cm) participated in this experiment. All participants signed an informed consent approved by the National Institutes of Health (NIH, Bethesda, MD, USA) Institutional Review Board (IRB) prior to the experiment.

First, two passive markers were placed on the skin over the posterior superior iliac spine for pelvis tracking and two more markers were placed on the lateral side of each foot at a distance of 2 cm below the ankle joint for foot tracking. The two foot markers were used to measure the positions of the subject’s feet for data analysis. Next, the subjects were instructed to walk freely overground to obtain their preferred speed (*V_pref_*) using Mocap. Based on their preferred speed, their slow target speed (75% of *V_pref_* and fast target speed (125% of *V_pref_*) were defined to design the acceleration/deceleration in TW. Prior to TW, the subjects were asked to tuck in shirts to reduce the amount of noise in torso position measured by the Xtion sensor. Then, they walked on the treadmill for approximately 1 min with the slow/fast target speeds displayed on the PC monitor in order to get accustomed to TW as well as the visual biofeedback.

The subjects walked on the two ITMs (the proposed ITM and the typical ITM) presented in random order. In each TW test, they were asked to quickly accelerate/decelerate their walking speed from the slow to the fast target speed and from the fast to the slow target speed. It is noteworthy that this instruction corresponds to the characteristic of natural acceleration in OW [26]. Due to the visual biofeedback, the subjects could match their walking speed to the target speeds. Between the acceleration/deceleration periods, the subjects were asked to maintain the two target speeds for approximately 15 s. After several practice periods, five acceleration and deceleration trials were performed for each subject with each ITM.

Before starting the walking trials on the ITM, subjects were informed that a hand raise would stop the ITM. After all acceleration/deceleration trials were completed, subjects were asked to use the “hand raise” gesture to come to a complete and safe stop. In order to maintain blinding, the stop command was manually implemented for the typical ITM when the subject made the hand raise gesture. After finishing TW with the first ITM, the subjects were allowed to rest for approximately 2 min before starting with the second ITM. The subjects walked on each ITM for 3 min on average. For each ITM, subjects provided a rating from 1 (worst) to 10 (best) that represented similarity to OW and scored the perceived level of safety for the gesture-based stopping method.

### 3.3. Data Analysis

**Figure 6:**
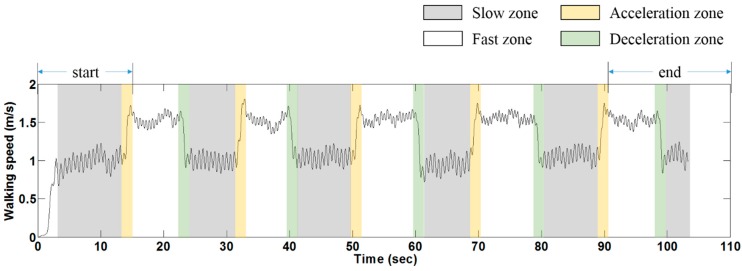
A typical walking speed profile and zones of treadmill walking on the proposed ITM in this experiment.

According to the walking speed profile, the TW on each ITM was classified into four zones (fast, slow, acceleration, and deceleration), and each zone of the two ITMs was compared for performance. Figure 6 demonstrates a typical walking speed profile and illustrates how the zones were determined. Of these, the fast zone and the slow zone are used to represent steady-speed walking, while acceleration and deceleration represent transient walking. Note that the start period (the first slow and acceleration zones) and the end period (the last fast/slow and deceleration zones) were excluded from the data analysis (Figure 6). The time durations of the acceleration/deceleration zones (up to 2 s) were much shorter than the fast/slow zones (up to 18 s) due to the instruction we gave (Figure 6).

To analyze steady-speed walking on ITMs, the following spatial and temporal gait parameters in the fast/slow zone were utilized: walking speed, step length, and cadence. The step length was obtained as the distance between the left foot and right foot in the anteroposterior axis during double-stance phase. The positions of the feet in the superoinferior axis measured by Mocap were used to detect the double-stance phase. The cadence was calculated based on the number of steps per minute. Transient walking on ITMs was analyzed using the subject’s mean acceleration as well as the peak treadmill belt acceleration. The former parameter was computed using numerical differentiation of walking speed in each acceleration/deceleration zone in MATLAB (The Mathworks, Inc., Natick, MA, USA). It has been reported that the latter, peak treadmill belt acceleration, is proportional to the subject’s maximum instantaneous acceleration [6] and also represents the maximum size of anomalous force, which results in unnatural walking on an ITM, especially in transient walking [6].

A dependent (paired) samples *t*-test was performed to investigate the statistical difference between the two ITMs. After excluding the start and end period, each subject had 4 valid fast/slow/acceleration/deceleration zones (Figure 6). Hence, a total of 40 samples for each parameter and zone (*i.e.*, step length in the slow zone) were used for the *t*-test. Finally, the qualitative comparison of two ITMs as well as the qualitative evaluation of the stop function of the proposed ITM was conducted using a questionnaire.

## 4. Results

### 4.1. Preferred OW and Walking Performance on ITMs

The subjects’ preferred overground walking speeds ranged between 1.0 and 1.3 m/s (1.14 ± 0.10 m/s). All subjects were easily accustomed to both ITMs; they could control their walking speed using visual biofeedback provided within a one-minute practice period.

### 4.2. Steady-Speed Walking on ITMs

**Figure 7:**
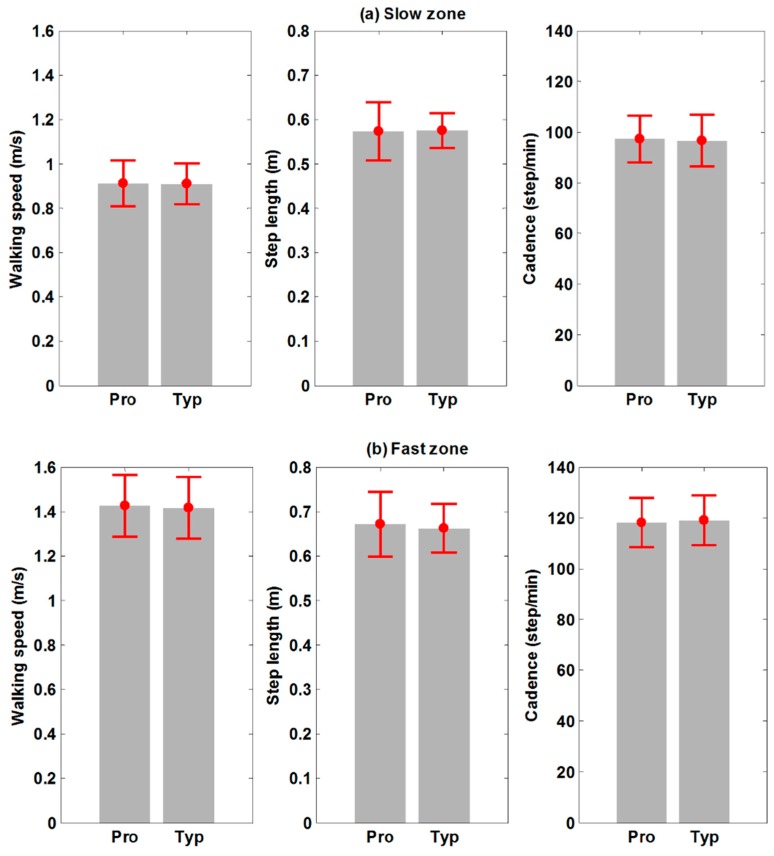
Quantitative comparison of three parameters at slow/fast zones with two ITMs (Pro: the proposed ITM; Typ: the typical ITM) (**a**) Slow zone; (**b**) Fast zone.

The three parameters for the slow/fast zone on ITMs, including the walking speed, the step length, and the cadence, are displayed in Figure 7. In both the slow and the fast zones, all parameters for the proposed ITM are not statistically different from those of the typical ITM, as shown in Table 3. This means that steady-speed walking on the proposed ITM is very similar to the typical ITM.

**Table 3:** Comparison of steady-speed walking on proposed and typical interactive treadmills.

		Proposed ITM	Typical ITM	*p*-Value
Walking speed (m/s)	slow (zone)	0.91 ± 0.10	0.91 ± 0.09	0.758
	fast	1.42 ± 0.14	1.42 ± 0.14	0.667
Step length (m)	slow	0.57 ± 0.07	0.58 ± 0.04	0.810
	fast	0.67 ± 0.73	0.66 ± 0.06	0.281
Cadence (step/min)	slow	97.38 ± 9.26	96.71 ± 10.20	0.603
	fast	118.18 ± 9.67	119.10 ± 9.82	0.251

### 4.3. Transient Walking on ITMs

**Figure 8:**
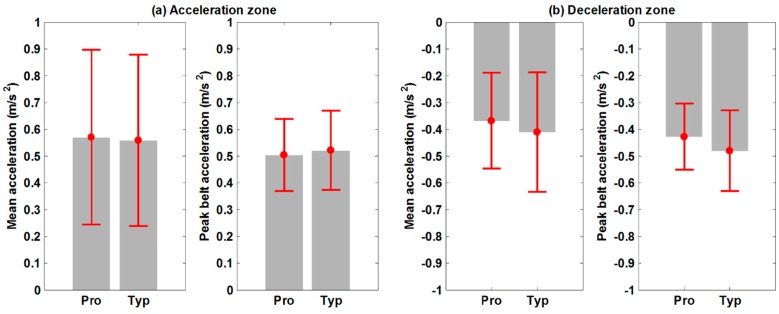
Quantitative comparison of two parameters at acceleration/deceleration zones with two ITMs (Pro: the proposed ITM; Typ: the typical ITM) (**a**) Acceleration zone; (**b**) Deceleration zone.

Figure 8 represents the subject’s mean acceleration and the peak treadmill belt acceleration in the acceleration/deceleration zone. The proposed ITM does not result in different accelerated walking when compared to the typical ITM in both the acceleration and deceleration zones (Table 4). Moreover, the peak belt accelerations of each ITM were not significantly different (Table 4).

**Table 4:** Comparison of transient walking on proposed and typical interactive treadmills.

		Proposed ITM	Typical ITM	*p*-Value
Subject’s mean acceleration (m/s^2^)	acceleration	0.57 ± 0.32	0.56 ± 0.32	0.843
	deceleration	0.37 ± 0.18	0.41 ± 0.22	0.078
Peak belt acceleration (m/s^2^)	acceleration	0.50 ± 0.14	0.52 ± 0.15	0.339
	deceleration	0.43 ± 0.12	0.48 ± 0.15	0.051

### 4.4. Evaluation by Questionnaire

**Figure 9:**
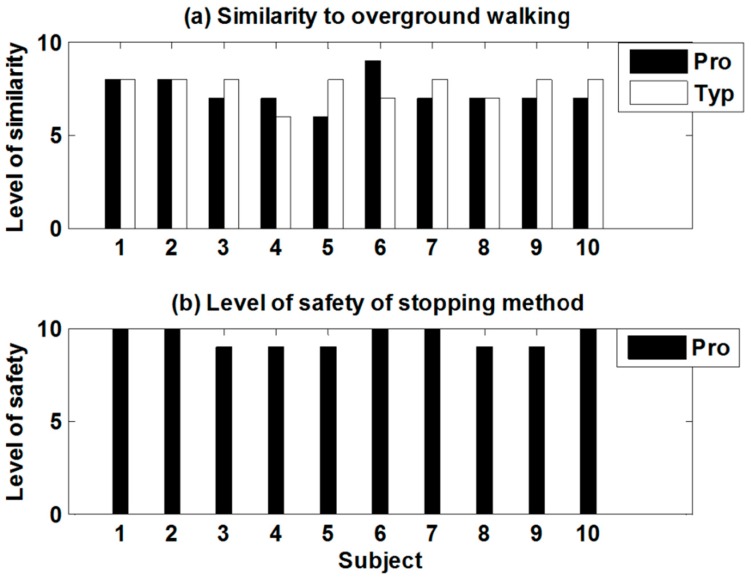
Results of qualitative feedback (Pro: the proposed ITM; Typ: the typical ITM). (**a**) Level of similarity to overground walking; (**b**) Level of safety of developed stopping method.

For qualitative comparisons, the level of similarity to OW and the level of safety of the stopping method, are displayed in Figure 9. Regarding the level of similarity, the proposed ITM (7.3 ± 0.8) is slightly worse than the typical ITM (7.6 ± 0.7), but the difference is not significant (Figure 9a). As shown in Figure 9b, all subjects gave positive feedback on the stopping method of the proposed ITM (9.5 ± 0.5).

## 5. Discussion

Even though the proposed ITM does not use an expensive and high performance sensor-based system, such as a Mocap with an instrument treadmill, the experimental results show that there is no difference between TW on the proposed ITM when compared to a typical ITM. According to the specification of the sensor systems, the order of random error magnitude of the Mocap (less than 1 mm) was about 100 times smaller than that of the Xtion sensor (up to 13 mm at 3 m distance [25]) and the sampling rate of the Mocap (120 Hz) was about 4 times faster than the Xtion (30 Hz). However, the significant difference in sensor performance does not necessarily affect the performance of ITM. This implies that the expensive and accurate Mocap system is over a specification required for implementing ITM provided that the appropriate high-level control scheme is selected. As shown in Figure 8, the control scheme used reduces the required belt acceleration (below 0.6 m/s^2^ on average) relative to the conventional PID [6,13]. Therefore, the inexpensive treadmill in the proposed ITM is acceptable for implementation of an ITM.

In the four zones of TW, the only noticeable difference between the two ITMs appeared in the deceleration zone. For the proposed ITM, the subject’s mean acceleration and peak belt acceleration are both slightly smaller than for the typical ITM (Figure 8b). The difference is not statistically significant, but the *p*-values obtained from the *t*-test were relatively small (*p* = 0.078 for the subject’s mean acceleration; *p* = 0.051 for the peak belt acceleration; see Table 4). This result raises the possibility that in comparison to the typical ITM, some walkers on the proposed ITM would be less likely to make rapid decelerations. This could be due to the relatively large latency of the proposed ITM, which results from the slow sampling rate of the motion sensor and the inexpensive treadmill. It is possible that the latency causes the walker to feel unstable during rapid changes in walking speed on the proposed ITM and that feeling could be exacerbated during deceleration due to the lack of rear vision during walking. In Figure 8, the standard deviations of the kinematic parameters during transient walking were greater than those from steady-speed walking due to variation in individual performance to accelerate/decelerate quickly.

The feedback obtained from the questionnaire is consistent with the results of the quantitative comparison above. While both ITMs achieved similar results, a slight difference could result from the evaluation of deceleration. For example, one subject whose mean deceleration was minimal rated the lowest score on the proposed ITM. The feedback on the developed stopping method was positive. There was no failure in attempting to stop and none of the subjects grasped the handrail of the treadmill during the experiment.

Since the Xtion is a low-cost infrared-type motion sensor, we expect that the total cost for implementing an ITM can be significantly reduced. The use of the sensor also adds convenience to the proposed ITM. In contrast to the existing ITMs, the proposed ITM does not require subjects to have markers attached or to put on a harness or the use of a force sensor, potentiometer, or ultrasonic range finder. Another benefit of the sensor is the gesture-based stopping method that improves both convenience and safety of the ITM. This improved cost-effectiveness and convenience would increase accessibility of the proposed ITM. For instance, these improvements enable the clinician to start to use an ITM faster and to simplify the clinical implementation process for an ITM.

However, there are potential drawbacks of using the low-cost infrared-type motion sensor. To visualize the subject’s entire body, the Xtion sensor was located 2 m behind the endpoint of the treadmill. This increases the space required for whole system setup which might be a constraint for small hospitals or local clinics. In addition, the robustness of skeleton recognition algorithm may affect safety of the system when there are multiple persons within the view angle of the sensor system. To our knowledge, the Microsoft Kinect V2 (Microsoft Corporation, Redmond, WA, USA) which is more recent device would improve the robustness.

This paper is limited in that the proposed ITM was implemented by simulating an inexpensive treadmill through reducing the performance of an instrumented treadmill. Even though this experimental design aided in producing a fair comparison for this study, the platform of the future ITM implementation should be an actual inexpensive treadmill. The kinematic parameters obtained during transient walking had greater variation due to individual’s performance of quick acceleration/deceleration was different each other. However, large variation would result in less reliable statistical analysis. For more reliable statistics, more data need to be collected. Another limitation is that this study used only kinematic gait parameters in a small healthy population. More analyses on kinematics as well as kinetics in TW with the proposed ITM, and with larger populations, are needed to evaluate performance with respect to simulating OW. Future plans include optimizing the stopping method developed with various gesture candidates for broader use and combining the proposed ITM with 3-D virtual reality [9,27] to minimize effects of the absence of visual flow that remains unsolved to date [5].

## 6. Conclusions

A low-cost infrared motion sensor was used to build a low-cost and convenient version of an interactive treadmill has been proposed for widespread use, such as increasing accessibility of the ITM in local clinics. The ASUS Xtion sensor was employed and by choosing the most reliable landmark, we found that the sensor could provide the subject’s position with acceptable accuracy. Thanks to the gesture recognition function provided by the sensor system, a convenient and safe stopping method was developed for this version of an interactive treadmill. In addition, a novel belt speed control scheme was applied for implementing this interactive treadmill without degrading performance with respect to simulating overground walking. A pilot experiment with ten healthy subjects showed that the proposed interactive treadmill has comparable performance to a typical interactive treadmill that is based on a more expensive and less convenient setup, and verified the benefit of using the new gesture-based stopping method.
